# Inflammatory markers and occurrence of falls: Bambuí Cohort Study of Aging

**DOI:** 10.11606/S1518-8787.2019053000855

**Published:** 2019-03-26

**Authors:** Juleimar Soares Coelho de Amorim, Karen Cecília Lima Torres, Andréa Teixeira-Carvalho, Olindo Assis Martins, Maria Fernanda Lima-Costa, Sérgio Viana Peixoto

**Affiliations:** I Instituto René Rachou. Fundação Oswaldo Cruz. Fiocruz Minas. Programa de Pós-Graduação em Saúde Coletiva. Belo Horizonte, MG, Brasil; II Instituto René Rachou. Fundação Oswaldo Cruz. Fiocruz Minas. Grupo Integrado de Pesquisas em Biomarcadores. Belo Horizonte, MG, Brasil; III Instituto René Rachou. Fundação Oswaldo Cruz. Fiocruz Minas. Núcleo de Estudos em Saúde Pública e Envelhecimento. Belo Horizonte, MG, Brasil; IV Universidade Federal de Minas Gerais. Escola de Enfermagem. Departamento de Enfermagem Aplicada. Belo Horizonte, MG, Brasil

**Keywords:** Aged, Aging, Accidental Falls, Risk Factors, Biomarkers, Inflammation Mediators, Idoso, Envelhecimento, Acidentes por Quedas, Fatores de Risco, Biomarcadores, Mediadores da Inflamação

## Abstract

**OBJECTIVE:**

Analyze whether inflammatory markers are associated with falls among older adults living in Bambuí.

**METHODS:**

Study that analyzed baseline data from a Bambuí Cohort Study of Aging, involving 1,250 participants. Data about falls were collected from previous 12 months, classified as single or multiple occurrence and severity (participant seeking health services). Information about sociodemographic characteristics, health behaviors and health condition was also collected and used as confounding factors. The exposures of interest included interleukins (IL-1β, IL-6, IL-8, IL-10, IL-12), tumor necrosis factor (TNF), ultra-sensitive C-reactive protein (us-CRP) and chemokines (CXCL9, CCL5, CCL10, MCP1). Data were processed through logistic regression, obtaining odds ratio and 95% confidence interval (95%CI).

**RESULTS:**

The prevalence of falls was 27.1%; 40.1% of the older adults reported multiple falls and 33.3% sought health services. After adjustments, the following elevated levels were associated with falls: us-CRP (OR = 1.46, 95%CI 1.04–2.03), CCL5 (OR = 1.38, 95%CI 1.01–1.90) and CXCL9 (OR = 1.43, 95%CI 1.02–2.02). An association was observed between the number of elevated markers and the occurrence of falls: two (OR = 1.47, 95%CI 1.02–2.12) and three (OR = 2.08, 95%CI 1.12–3.87) elevated biomarkers indicated fall probability of 32.0% and 39.4%, respectively.

**CONCLUSIONS:**

Elevated levels of us-CRP, CCL5 and CXCL9, which were associated with falls, may contribute to a proper understanding of the mechanism associated with the occurrence of falls among older people.

## RESUMO

**OBJETIVO:**

Analisar se marcadores inflamatórios estão associados a quedas em idosos vivendo na comunidade.

**MÉTODOS:**

Estudo da coorte de idosos de Bambuí, envolvendo 1.250 participantes da linha de base do projeto. Foram coletadas informações sobre quedas nos últimos 12 meses, classificadas quanto à ocorrência (única ou múltipla) e gravidade (procura por serviços de saúde). O inquérito também continha informações a respeito das características sociodemográficas, comportamentais e condições de saúde, as quais foram utilizadas como fatores de confusão. As exposições pesquisadas incluíram: interleucinas (IL-1β, IL-6, IL-8, IL-10 e IL-12), fator de necrose tumoral (TNF), proteína C reativa ultrassensível (PCRus) e quimiocinas (CXCL9, CCL5, CCL10 e MCP1). O tratamento dos dados foi realizado por meio de regressão logística, obtendo-se *odds ratio* e intervalo de 95% de confiança (IC95%).

**RESULTADOS:**

A prevalência de queda foi 27,1%; 40,1% dos idosos relataram quedas múltiplas e 33,3% procuraram serviços de saúde. Após ajustes, permaneceram associados às quedas os níveis elevados de PCRus (OR = 1,46; IC95% 1,04–2,03), CCL5 (OR = 1,38; IC95% 1,01–1,90) e CXCL9 (OR = 1,43; IC95% 1,02–2,02). Houve associação entre o número de marcadores elevados e a ocorrência de quedas: dois (OR = 1,47; IC95% 1,02–2,12) e três (OR = 2,08; IC95% 1,12–3,87) biomarcadores aumentados predisseram probabilidades de quedas iguais a 32,0% e 39,4%, respectivamente.

**CONCLUSÕES:**

Os níveis elevados de PCRus, CCL5 e CXCL9, que estiveram associados a quedas, podem contribuir para o adequado entendimento do mecanismo associado à ocorrência desse evento em idosos.

## INTRODUCTION

Falls represent an important health problem among older adults, with a significant impact due to their high prevalence and its consequences, such as hospitalization, disability and death^1 – 3^ . They are also the subject of extensive scientific investigations, either due to its multifactorial nature or its impact on the health of population, treatment expenses, and the identification of preventive and rehabilitative measures ^3^ .

Population studies estimate that 28 to 42% of older adults in Brazil are affected by falls at least once a year ^4^ , representing two-thirds of all accidents and a marker of risk for mortality, besides a strong predictor of functional limitation ^5^ . Many different characteristics are associated with fall, such as age (old age), place of residence (home), sex (female), geriatric syndromes (multiple morbidities, polypharmacy, frailty and sarcopenia), functional condition (limitation in daily activities, mobility and balance), specific diseases (diabetes, arthritis, pain, stroke, dementia and visual deficit), lifestyle (sedentary behaviors and alcoholism), and risk behaviors in household activities; however, inflammatory markers are still underexplored ^6^ ^,^ ^7^ .

The high degree of long-term inflammation associated with aging, called *inflammaging* ^8^ , has been addressed by researchers to understand adverse health outcomes^9–11^. Among the determinants of falls, high plasma levels of biomarkers are relevant – particularly interleukins and others such as tumor necrosis factor and C-reactive protein ^10^ ^,^ ^12^ ^,^ ^13^ . Although this relationship may occur due to the association of biomarkers with events preceding falls, such as functional decline, development of fragility, cognitive disorders and sarcopenia ^14^ ^,^ ^15^ , the degree of inflammation is also an early and sensitive predictor, even before the diagnosis of changes related to aging.

Due to the high cost of measuring these biomarkers, especially in population studies, and the complexity of determining *inflammaging* , data about inflammatory profiles among older people are often limited to a small subset of cytokines. Likewise, the challenge to understand the complexity of multiple causes of falls in older people has received greater attention from researchers. Therefore, studies on the association between an extensive range of inflammatory markers and the occurrence of falls among older people, considering other potential confounding factors, may contribute to the knowledge produced to date about chronic inflammation and this event. Considering the above, this study aimed to analyze whether inflammatory markers are associated with the occurrence of falls among older adults from the baseline of the Bambuí Cohort Study of Aging.

## METHODS

### Study Design and Population

The Bambuí Cohort Study of Aging is a population prospective study that is ongoing in Bambuí, Minas Gerais. The participants were identified in 1996 from a complete census conducted in the city. All 1,742 residents aged 60 years or older on January 1, 1997 were invited to participate in the study; 1,606 (92.2%) were interviewed and 1,496 (85.9%) were submitted to a physical examination and blood exam. Baseline data were used in this study, with collected blood frozen at -80°C. This study was approved by the FIOCRUZ Ethics Committee. All participants agreed to participate and signed an informed consent form.

### Dependent Variable

The dependent variable refers to falls reported in the 12 months preceding the baseline interview. The event was also characterized according to the number of occurrences in the previous year, resulting in the classification of single fall or multiple falls (two or more), and according to fall severity, as measured by participant seeking health services as a result of the event. This study uses the concept of fall from the Prevention of Falls Network Europe (ProFaNE) network: an unexpected event in which the participant comes to rest on the ground, floor, or lower level. This is a more appropriate definition for multicenter and/or population studies ^16^ .

### Explanatory Variables (Inflammatory Profile)

For biochemical and immunological exams, the participants were instructed to be on a fast for at least 12 hours. The blood samples were centrifuged, cooled and then sent to the Laboratory of Epidemiology and Medical Anthropology of the René Rachou Institute/FIOCRUZ in Belo Horizonte for analysis ^17^ ^,^ ^18^ . In 2015, from such frozen blood samples, serum dosages were performed for the following inflammatory markers: interleukins (IL-1β, IL-6, IL-10, IL-12), tumor necrosis factor (TNF), chemokines (CXCL8/IL-8, CXCL9, CCL2, CXCL10, CCL5), and ultra-sensitive C-reactive protein (us-CRP). For measurement, 5 mL of blood were collected in a vial containing sodium heparin using venipuncture and a vacuum collection system (Vacutainer, Becton Dickinson – BD).

Flow cytometry in multiplex assays (CBA immunoassay kit, Becton Dickinson Biosciences Pharmingen, USA) was used for the quantitative determination of cytokines (human inflammatory kit) and serum chemokines (human chemokine kit). The CBA inflammatory kit comprises microspheres coupled to monoclonal antibody (MoAb) against cytokines IL-1β, IL-6, TNF, IL-12 and IL-10, and the CBA chemokine kit detects CXCL8, CXCL9, CXCL10, CCL2 and CCL5. In the second step, anti-cytokine antibodies fluorescently labeled with phycoerythrin (PE) were used. Therefore, the concentration of individual cytokines was indicated by their fluorescent intensity. Data were obtained using a FACSVerse flow cytometer (Becton Dickinson, USA), while BD FCAP Array 3.0 (Becton Dickinson, USA) was used for sample analysis. Intra- and inter-assay coefficients of variation were 5%–10% and 7%-12%, respectively. Dosages of IL-6, CXCL8, CXCL9, CXCL10, CCL2 and CCL5 were expressed as medians and interquartile ranges. IL-1β, TNF, IL-12 and IL-10 showed very low detectable levels and were considered dichotomous variables (undetectable versus detectable).

### Confounding Factors

Confounding variables were selected considering previous studies on falls and biomarkers, including sociodemographic factors (age, sex and schooling), health behaviors, health conditions, and medication use ^7^ ^,^ ^19^ .

Interviews were conducted with the participants to collect information about alcohol consumption, smoking, physical activity level, history of stroke, infarction, arthritis/rheumatism, depression symptoms and possible cognitive impairment. These interviews were conducted by trained interviewers at the participant’s home with the participant or a close respondent whenever a health problem did not allow the participant to answer.

Alcohol consumption was considered a risk when the respondent reported seven or more amounts per week in the 12 months prior to the interview. Cards were displayed showing the amount of beer, wine or spirits, according to previous standardization ^17^ . In relation to smoking, participants were classified as “current smoker” if they had smoked at least 100 cigarettes in their lifetime and continued smoking at the moment of the interview. Information about physical activity was collected through a structured questionnaire about the type of activity, frequency and duration of practice in the last 90 days. The level of physical activity was calculated considering the level of oxygen consumed, quantified by energy expenditure in metabolic equivalents (METs). Only moderate and vigorous activities were considered, minimum duration of 10 minutes without interruption. Individuals of energy expenditure of less than 450 MET.min per week were classified as insufficiently active ^20^ . The history of stroke was assessed using standardized questionnaires (Plan and Operation of the Third National Health and Nutrition Examination Survey, 1988-1994, 1994); and reported infarction and arthritis or rheumatism were evaluated using the history of medical diagnosis for these conditions. The presence of depressive symptoms was evaluated using the General Health Questionnaire (GHQ-12), with a cut-off point ≥ 5 for the score obtained in this instrument. The Mini-Mental State Examination (MMSE) was used to assess the cognitive function, with a previously validated cut-off point for this population: a score lower than 22, corresponding to the lower quartile ^21^ . To evaluate the number of chronic diseases (none, one, two or more), the following conditions were included: hypertension, diabetes mellitus, arthritis, stroke, acute myocardial infarction, and depressive symptoms. Information regarding continuous medication was collected through evidence (prescriptions or packaging), and the simultaneous use of five or more medications was considered polypharmacy ^22^ .

A functional capacity was assessed by the domains of mobility and basic and instrumental activities of daily living (ADL). To analyze mobility limitation, the following tasks were selected: walking about two or three blocks; climbing stairs; stooping, squatting and kneeling; and lifting or carrying weight (5 kg). The activities of daily living evaluated were: meal cooking; managing money; shopping; cleaning; using public transportation; standing up when sitting on a chair without arms; lying down or getting out of the bed; eating; bathing; dressing; and toileting. Limitations in the two domains analyzed (ADL and mobility) were categorized into three levels (no or some difficulty, great difficulty and unable to perform).

Physical examination was performed at the study clinic by professionals trained and certified by the team and included the measurement of blood pressure, blood glucose, body weight and height. Hypertension was defined by systolic and/or diastolic blood pressure equal to or greater than 140 mmHg and 90 mmHg, respectively. Blood pressure was measured at least 30 minutes after the last dose of ingested caffeine or smoked cigarette, using a mercury sphygmomanometer (Tycos 5097-30, USA) and Littman Cardiology II stethoscope (USA). Three measurements were performed after five minutes at rest observing two-minute intervals. Diabetes mellitus was defined as a fasting blood glucose level of 126 mg/dL or greater. Drug treatment for both diseases was also considered a diagnosis. The anthropometric evaluation used the body mass index (BMI), calculated as the weight (kg) divided by the square of the height (m), with classification of individuals according to the standardization of Lipschitz ^23^ .

The fact that Bambuí was an endemic area for Chagas disease, which is caused by the protozoan *Trypanosoma cruzi* , was also considered a potential confounding variable in the analyses. The infection was defined by positive serology in three tests: the hemagglutination assay (Biolab Mérieux, Rio de Janeiro, Brazil) and two enzyme-linked immunosorbent assays (Elisa) (Abbott Laboratories Inc., Chicago, USA, and Wiener Laboratories, Rosario, Argentina).

### Statistical Analysis

Unadjusted analyses were based on the characteristics of the population, measured in proportions and mean values (with respective standard deviations) and used to compare the groups that reported falls or no falls. Pearson’s chi-square test was used to compare proportions, including detectable levels of categorized inflammatory markers (IL-1β, TNF, IL-12, and IL-10); for mean values, the Student’s t-test was used. For the levels of IL-6, CXCL8, CXCL9, CXCL10, CCL2 and CCL5, the comparison between the groups was performed by the Mann-Whitney test, considering the median of the distribution in each group.

Logistic regression was used to estimate odds ratios and respective confidence intervals (95%), without adjustment and with progressive adjustment, including sociodemographic variables (Model 1), adding the variables of health behaviors (Model 2), and adding to the previous model the variables of health condition and use of medication (Model 3). This analysis was performed for all markers included in the study, using the interquartile range distribution, that is, categorizing them in the 25^th^ and 75^th^ percentiles (IL-6, CXCL8, CXCL9, CXCL10, CCL2 and CCL5) to detect the possible dose-response relationship in association with falls, or considering detectable levels (IL-1β, TNF, IL-12 and IL-10).

Statistically associated biomarkers (p < 0.05) with reported falls in the final model were selected to constitute the inflammatory profile of the older adults, combining the number of altered markers with intermediate or higher interquartile ranges. For this, the logistic regression model was adjusted and the probability of occurrence of falls for each older person was calculated according to the number of altered markers, keeping the adjustment for all the confounding factors considered. All statistical models of logistic regression were suitable at the Hosmer-Lemeshow goodness of fit test. All analyses were performed in Stata 13.0 software, considering the significance level of 5%.

## RESULTS

A total of 1,250 older people was included in this cohort baseline analysis, whose records contained all information considered in this study. The prevalence of falls was 27.1%, of which 40.1% were multiple falls and 33.3% sought health services.

Table 1 describes the characteristics of the studied population and the association with falls. Among the participants, whose mean age was 68.8 (SD = 6.9) years, 61.7% were female, 17.3% were current smokers, 4.7% consumed alcohol, 27.3% were unable to perform ADL, 25.5% had mobility limitations, and 22.6% were classified as polypharmacy. Considering the unadjusted association with falls, these covariates presented a significant difference among the older people who reported this event.

**Table 1:** Sociodemographic conditions, health behaviors, health conditions and use of medication, according to reported falls in the last year; baseline data of Bambuí Cohort Study of Aging.

Characteristics	Total	Falls		p^a^

		No	Yes	
		
	(n = 1,250)	(n = 911)	(n = 339)	
Sociodemographic condition				

Age (years) (mean and standard deviation)	68.8 (6.9)	68.4 (6.7)	69.8 (7.4)	0.001
Female	61.7	56.6	75.2	< 0.001
Schooling < 4 years	63.2	62.5	64.5	0.743

Health behaviors				

Current smoker	17.3	18.4	14.2	0.001
Alcohol consumption (≥ 7 amounts/week)	4.7	5.2	3.5	0.001
Insufficiently active	26.8	26.0	28.9	0.305

Health condition and medication use				

Nutritional status (BMI)				0.210
Well nourished (18–22 kg/m^2^)	39.7	40.9	36.3	
Underweight (< 18 kg/m2)	27.8	27.9	27.7	
Overweight (> 22 kg/m^2^)	32.5	31.2	36.0	
Number of chronic diseases^b^				0.130
One	35.4	33.9	39.5	
Two or more	46.7	47.3	45.1	
Positive serology for *T. cruzi*	37.0	36.0	39.5	0.251
Cognitive impairment (MMSE < 22)	22.1	20.8	25.7	0.062
Limitation in ADL				< 0.001
None or some	52.7	56.3	43.1	
Large	20.0	20.4	18.9	
Unable to perform	27.3	23.3	38.1	
Limitation in mobility				0.001
None or some	57.9	60.0	52.2	
Large	16.6	17.3	14.5	
Unable to perform	25.5	22.6	33.3	
Polypharmacy (use of 5 medications or more)	22.6	21.0	26.8	0.027

BMI: body mass index; *T. cruzi*: *Trypanosoma cruzi* ; MMSE: Mini-Mental State Examination; ADL: activities of daily living

Note: Values expressed in percentages, except when specified.

^a^ Student’s t-test or Pearson’s chi-square test.

^b^ Hypertension, diabetes mellitus, arthritis or rheumatism, stroke, acute myocardial infarction and depression symptoms.

Table 2 describes the distribution of inflammatory markers in the total population and in relation to falls. The median of serum levels of us-CRP, CXCL10 and CXCL9 was higher in the group reporting a fall in the previous year.

**Table 2:** Distribution of inflammatory markers in relation to the occurrence of falls in the last year; baseline data of Bambuí Cohort Study of Aging.

Inflammatory markers	Total	Falls		p*

		No	Yes	
IL-1β (> 0.01 pg/mL), %	22.6	21.8	24.5	0.321
IL-6 (median and IQR)	1.0 (0.4–2.1)	1.0 (0.4–2.0)	1.2 (0.5–2.2)	0.121
TNF (> 0.02 pg/mL), %	17.5	16.5	20.4	0.108
IL-12 (> 0.02 pg/ml), %	7.8	7.2	9.1	0.264
IL-10 (> 0.01 pg/mL), %	42.6	42.8)	41.9	0.769
CXCL8 (median and IQR), pg/mL	3.0 (1.5–5.4)	2.9 (1.6–5.4)	3.0 (1.7–5.6)	0.268
CXCL9 (median and IQR), pg/mL	2,118.9 (1,231.0–4,141.8)	2,156.7 (1,145.7–3,804.1)	2,851.8 (1,465.0–5,104.1)	< 0.001
CXCL10 (median and IQR), pg/mL	3,026.8 (1,982.4–4,712.2)	2,906.6 (1,939.3–4,489.8)	3,318.1 (2,145.6–5,204.9)	0.017
CCL2 (median and IQR), pg/mL	38.4 (25.3–56.8)	39.0 (25.4–56.6)	37.1 (24.3–57.9)	0.697
CCL5 (median and IQR), pg/mL	863.1 (547.6–1,612.4)	831.9 (527.0–1,598.7)	950.5 (594.2–1,712.4)	0.055
us-CRP (median and IQR), mg/mL	3.2 (1.4–6.5)	3.1 (1.3–6.1)	3.9 (1.6–7.6)	0.003

TNF: tumor necrosis factor; us-CRP: ultra-sensitive C-reactive protein; IQR: interquartile range

* Mann-Whitney test for comparison between median values or chi-square test for comparison between proportions.

Table 3 shows the associations between reported falls and inflammatory markers, both unadjusted and adjusted for sociodemographic conditions, health behaviors, health condition, and use of medication. Considering the complete adjustment (Model 3), the highest levels of us-CRP (OR = 1.46, 95%CI 1.04–2.03), CCL5 (OR = 1.38, 95%CI 1.01–1.90) and CXCL9 (OR = 1.43, 95%CI 1.02–2.02) were associated with reported falls. Higher levels of IL-6 and CXCL10 were associated with the event only in the unadjusted analysis.

**Table 3:** Association between reported falls in the last year and inflammatory markers; baseline data of Bambuí Cohort Study of Aging.

Inflammatory markers	Odds ratio (95% confidence interval)			

	Unadjusted	Model 1	Model 2	Model 3
IL-1β^a^	1.16 (0.87–1.56)	1.10 (0.82–1.49)	1.09 (0.80–1.47)	1.09 (0.79–1.50)
IL-6^b^ in pg/mL (ref.: < 0.45)				
0.45–218	1.28 (0.94–1.74)	1.19 (0.87–1.63)	1.19 (0.87–1.63)	1.17 (0.84–1.62)
> 2.18	1.40 (1.02–1.91)	1.34 (0.98–1.85)	1.35 (0.98–1.87)	1.30 (0.93–1.80)
TNF^a^	1.30 (0.94–1.78)	1.32 (0.79–1.34)	1.30 (0.94–1.80)	1.23 (0.88–1.72)
IL-12^a^	1.29 (0.82–2.01)	1.32 (0.79–1.34)	1.31 (0.83–2.07)	1.19 (0.74–1.90)
IL-10^a^	0.96 (0.75–1.24)	0.95 (0.74–1.23)	0.95 (0.73–1.23)	0.96 (0.74–1.25)
CXCL8^b^ in pg/mL (ref.: < 1.6)				
1.6–5.66	1.31 (0.97–1.78)	1.26 (0.93–1.73)	1.25 (0.92–1.71)	1.29 (0.94–1.77)
> 5.66	1.24 (0.91–1.69)	1.22 (0.88–1.68)	1.20 (0.87–1.65)	1.19 (0.85–1.65)
CXCL9^b^ in pg/mL (ref.: < 1,231.86)				
1,231.86–4,279.89	1.23 (0.90–1.69)	1.21 (0.88–1.67)	1.21 (0.88–1.68)	1.23 (0.89–1.71)
> 4,278.89	1.68 (1.24–2.29)	1.46 (1.05–2.02)	1.45 (1.05–2.02)	1.43 (1.02–2.02)
CXCL10^b^ in pg/mL (ref.: < 1,984.25)				
1,984.25–4,847.78	1.27 (0.93–1.73)	1.14 (0.83–1.57)	1.14 (0.82–1.57)	1.14 (0.82–1.58)
> 4,847.78	1.58 (1.16–2.16)	1.35 (0.98–1.85)	1.35 (0.98–1.86)	1.36 (0.98–1.89)
CCL2^b^ in pg/mL (ref.: < 25.41)				
25.41–57.56	0.84 (0.62–1.14)	0.86 (0.63–1.17)	0.85 (0.63–1.16)	0.89 (0.65–1.23)
> 57.56	0.95 (0.70–1.29)	0.95 (0.69–1.29)	0.95 (0.70–1.30)	0.93 (0.68–1.28)
CCL5^b^ in pg/mL (ref.: < 553.37)				
553.37–1,622.97	1.13 (0.83–1.53)	1.26 (0.92–1.73)	1.25 (0.91–1.72)	1.26 (0.91–1.74)
> 1,622.97	1.23 (0.91–1.67)	1.39 (1.01–1.90)	1.39 (1.01–1.90)	1.38 (1.01–1.90)
us-CRP^b^ in mg/L (ref.: < 1.42)				
1.42–6.7	1.24 (0.91–1.70)	1.23 (0.89–1.70)	1.24 (0.90–1.72)	1.20 (0.86–1.67)
> 6.7	1.62 (1.19–2.21)	1.54 (1.12–2.10)	1.55 (1.13–2.13)	1.46 (1.04–2.03)

TNF: tumor necrosis factor; us-CRP: ultra-sensitive C-reactive protein; ref.: reference value

Model 1: adjusted for sociodemographic condition; Model 2: adjusted for sociodemographic condition and health behaviors; Model 3: adjusted for sociodemographic condition, health behaviors, health conditions and use of medication.

^a^ Detectable levels (> 0.01 for IL-1β and IL-10; > 0.02 for IL-12 and TNF), *versus* undetectable levels.

^b^ Interquartile range of distribution (categorized in 25^th^ and 75^th^ percentiles).

Table 4 shows an association between the number of elevated markers (above the third interquartile range) and the occurrence of falls, considering those with a significant association with the outcome in the previous analysis (us-CRP, CCL-5 and CXCL9). This analysis showed that two (OR = 1.47, 95%CI 1.02–2.12) and three (OR = 2.08, 95%CI 1.12–3.87) markers at their highest levels were associated with the occurrence of falls. The predicted probability of fall increases as one, two, or three altered markers increases above the third interquartile range at 25.0% (95%CI 21.0–29.0), 32.0% (95%CI 27.0–37.0) and 39.4% (95%CI 27.0–52.0), respectively.

**Table 4:** Association between reported falls in the last year and number of markers showing increased levels (above the third interquartile range); baseline data of Bambuí Cohort Study of Aging.

Number of markers showing increased levels	Odds ratio (95% confidence interval)			

	Unadjusted	Model 1	Model 2	Model 3
None	1.00	1.00	1.00	1.00
One	1.15 (0.85–1.56)	1.08 (0.79–1.49)	1.08 (0.79–1.48)	1.02 (0.74–1,41)
Two	1.70 (1.21–2.39)	1.57 (1.10–2.23)	1.57 (1.10–2.24)	1.47 (1.02–2.12)
Three	2.59 (1.44–4.68)	2.29 (1.25–4.19)	2.29 (1.25–4.20)	2.08 (1.12–3.87)

Model 1: adjusted for sociodemographic condition; Model 2: adjusted for sociodemographic condition and health behaviors; Model 3: adjusted for sociodemographic condition, health behaviors, health conditions and use of medication.

## DISCUSSION

In this population, falls were more frequent among older people with increased levels of us-CRP, CCL5 and CXCL9, after careful adjustment for factors that could confuse this association. The inflammatory profile analysis shows increased probability of falls according to the number of biomarkers at higher levels, reinforcing the relationship between falls and inflammation, a hypothesis that guided this study. This study demonstrated the inflammatory profile of older people, characterized by changes in three markers, was an important measurement to understand the association with falls, which is consistent with the phenomenon of *inflammaging* . However, it should be noted that such characterization included markers that are underexplored in the literature, such as CXCL9 and CCL5, besides CRP. Most studies analyze each biomarker in isolation; however, it is necessary to understand the inflammatory profiles, since no particular biomarker is able to track the multiplicity of etiologies, phenotypes and pathophysiology, especially in older people, with complex events and multiple causes ^6^ . Then, this combination of inflammatory markers may show the result of distinct pathogenic processes and complement the interpretation of the associations found in the analyses.

Although the results of specific markers observed in this study have not been reported in other populations, these associations are consistent with evidence demonstrating the importance of inflammatory mechanisms that increase the risk for physical decline, cognitive impairment, sarcopenia, frailty, cardiovascular and neurological diseases and articulation disorders^9,10,12,19,24–26^, which are events strongly associated with falls among older people. In this sense, these risk factors are considered mediators between inflammation and falls, producing catabolic effects on muscles and the nervous system. In this study, the association of inflammation with falls was strong, even after adjustment for variables that evaluated, even indirectly, these mediators. Then, while inflammation may lead to falls due to its link with traditional risk factors already described in the literature, other unexplored mechanisms also seem to contribute to the occurrence of this event and deserve further investigation. Therefore, the association between fall and the triad of us-CRP, CCL5 and CXCL9 reveals new markers associated besides the traditional ones.

The consistent association observed in this study suggests that elevated plasma levels of these biomarkers have an effect on falls, regardless of the diagnosis of other conditions that could mediate it. This result agrees with the hypothesis proposed by Esch ^13^ that inflammation is a common denominator of or triggers different pathophysiological processes, suggesting that the degree of inflammation is also important in understanding the determinants of falls. In general, perhaps due to the scarcity of studies analyzing a greater diversity of biomarkers, the empirical evidence shows the triad of CRP, IL-6 and TNF as the main ones involved in changes related to aging and its consequences ^27^ , such as physical decline, reduced muscle strength, synovial inflammation in the hips or knees and altered gait among older people. Then, induction of T cell chemotaxis and inflammatory responses could increase the risk of falls, since those physical and functional measurements are predictive of this outcome ^15^ .

Considering the triad of biomarkers that remained associated with falls after adjustment for confounding factors, the higher levels of us-CRP should be highlighted, which are classically described as part of *inflammaging* . Previous studies characterized this biomarker as a participant in systemic inflammation, responsible for the overall physical and functional decline of older people ^15^ , in agreement with the understanding of falls not only as an accidental occurrence ^2^ . In addition, the results of this investigation reinforce the hypothesis of an independent effect of elevated levels of this marker on the occurrence of falls, not only through a pathway mediated by other outcomes such as sarcopenia, frailty and chronic diseases ^6^ ^,^ ^9^ ^,^ ^12^ ^,^ ^15^ .

The exact mechanism of the causal relationship between increased levels of CXCL9 and CCL5 and the occurrence of falls among older people has not been clearly defined. This uncertainty can be attributed to the complexity of this class of inflammatory biomarkers given its redundant (multiple cytokines have the same functional effects) and pleiotropic function (action on different cell types and biological effects), as well as the complex etiology of falls. Despite that, these chemokines do not induce each other, but other mediators that are present in severe trauma and specific tissues, such as synovial fluid, extending the chronic inflammatory activity and mobilizing calcium to participate in muscle contraction and bone metabolism ^15^ ^,^ ^28^ . However, this study included a number of confounding factors, such as chronic diseases that are also associated with these biomarkers, physical performance and falls, which suggests independence of the association between the event and the inflammation evaluated through these chemokines.

Regarding the levels of IL-6, perhaps one of the most explored markers in studies on aging, the results of this study demonstrated an association with falls only in the unadjusted analysis, which was lost with the age adjustment. This result agrees with a study conducted by Verghese et al. ^29^ , who did not identify a statistically significant association between falls and this cytokine. These authors suggest levels of oxidative stress as determinants of falls. Although elevated levels of IL-6 are associated with functional limitation, gait dysfunction, cognitive impairment, and frailty ^10^ ^,^ ^11^ ^,^ ^15^ , our results do not show a direct relationship between this biomarker and falls. In this case, oxidative stress also appears as a possible mechanism that causes falls, regardless of age, sex, schooling and multiple morbidities ^29^ . However, a previous study suggested that pro-inflammatory biomarkers are associated with increased oxidative stress in healthy older people, which reinforces the hypothesis of concomitant falls among older people ^30^ .

The systematic use of biomarkers, especially inflammatory biomarkers, in older population is still incipient, given the high costs and complex laboratory analysis procedures involved. The results of this study contribute to a more detailed understanding of the association between the levels of these biomarkers and the occurrence of falls, supporting the development of future studies, which should consider a greater diversity of inflammatory markers. Scientific evidence has indicated that better public health conditions and collective interventions resulting in reduced inflammation in early life may lead to reduced morbidity caused by chronic conditions in older people ^13^ ^,^ ^14^ ^,^ ^27^ ^,^ ^29^ , which may also be considered for the occurrence of falls.

The limitations of this study include its sectional design, which does not allow to set a temporal relation between the variables, and the information bias, due to the difficulty of older people to remember occurrences of fall in the last year, which does not seem to have influenced the estimates, considering the prevalence found in this study was similar to that of other studies. On the other hand, this analysis was conducted in a population study with a high response rate, information collected through standardized instruments and trained professional interviewers, which ensured data quality, besides having included a wide range of biomarkers and confounding factors. Then, this study adds to current knowledge evidence of new biomarkers associated with this outcome.

In summary, our findings showed that, in a Brazilian population of older people, elevated levels of us-CRP, CCL5 and CXCL9, and the inflammatory profile based on these markers, are associated with the occurrence of falls, regardless of sociodemographic conditions, health behaviors, health conditions and use of medication. This evidence may help understand the mechanism involved in the occurrence of this event, supporting the planning of actions for individual or collective monitoring of the population with greater vulnerability to this outcome, at least regarding the inflammation profile of this population.
